# Case report: Peroral endoscopic myotomy for acute pandysautonomia-associated distal esophageal spasm in a child

**DOI:** 10.3389/fped.2022.935915

**Published:** 2023-01-17

**Authors:** Hanhua Zhang, Biyun Chi, Fengfan Wang, Pei Shao, Huanyu Liu, Ying Fang

**Affiliations:** ^1^ Department of Gastroenterology, Xi'an Children's Hospital, Xi ‘an, China; ^2^ Xi'an Medical University, Xi'an, China

**Keywords:** children, acute pandysautonomia, distal esophageal spasm, POEM, rare case

## Abstract

Acute pandysautonomia-associated distal esophageal spasm is a rare disease with an unclear etiology. Here, we describe a 12-year-old boy with an acute pandysautonomia-associated distal esophageal spasm who was treated using a peroral endoscopic myotomy (POEM). The patient's clinical features included recurrent dysphagia, nausea, vomiting, growth retardation, and signs of autonomic nerve dysfunction (e.g., a decreased production of tears and sweat, and an increased production of saliva). Signs of the distal esophageal spasm were visible in upper gastrointestinal radiography, endoscopy, and high-resolution esophageal manometry. After the POEM, the patient exhibited improvements in nausea and vomiting, and his dysphagia symptoms were relieved by the 6-month follow-up visit. However, the patient's neurological problems persisted. The satisfactory short-term clinical responses in our patient suggest that POEM is feasible, safe, and effective for the treatment of acute pandysautonomia-associated distal esophageal spasms in children.

## Introduction

Acute pandysautonomia is an acute or subacute onset disease that mostly occurs in young and middle-aged adults; there are few reports of affected children (1, 2). Symptoms in the digestive tract (e.g., unexplained nausea, vomiting, and/or dysphagia) may appear at the onset of the disease. Despite symptomatic treatment, there is a minimal improvement (3) and affected patients exhibit severe malnutrition, leading to cachexia. Currently, intravenous immunoglobulin and plasma exchange are the most common treatments for acute pandysautonomia (4).

Distal esophageal spasm (DES) is a rare condition that affects esophageal motility (5). The pathophysiology of DES reportedly involves an impairment in esophageal inhibition, which leads to premature contractions in the distal muscularis propria (6). Patients usually present with clinical manifestations of dysphagia, regurgitation, heartburn, and/or chest pain (7, 8). The diagnosis is supported by modalities such as a barium swallow and esophagogastroduodenoscopy examinations, and the final diagnosis can be confirmed by high-resolution esophageal manometry (HREM) (9). Although there is no standard treatment for DES, peroral endoscopic myotomy (POEM) is a less-invasive approach that improves symptoms in adult patients (10, 11), with a significant short-term effect. Because of the limited information concerning the use of POEM in children with DES, there is a need for reports describing such a treatment.

Here, we describe a 12-year-old boy with acute pandysautonomia-associated DES who had severe gastrointestinal symptoms and underwent POEM.

## Case description

A 12-year-old boy presented with recurrent nausea, vomiting, and dysphagia, as well as progressive symptoms of autonomic dysfunction. His family history was unremarkable and he had no previous history of similar symptoms.

Nineteen months before the present admission, the patient showed signs of pandysautonomia, including an atonic bladder, alacrima, and impaired sweating after a gastrointestinal illness with unclear etiology. At that time, an abdominal ultrasound, computed tomography, and gastrointestinal angiography had revealed superficial gastritis and duodenal bulbitis with reduced gastric and duodenal peristalsis. The patient had been treated with antacids, antiemetics, and anti-infectives for 2 months. However, his symptoms did not significantly improve.

Twelve months before the present admission, the patient began to vomit after eating because of worsening dysphagia. Physical examination revealed impaired sweating and salivation, mild pupillary dilation, and urinary retention. Cerebrospinal fluid, electromyography, and serum immunoglobulin M tests showed no abnormalities. Thoracic, lumbar, and sacral magnetic resonance imaging showed nerve root enhancement (L1 and L2 segments), with slightly greater enhancement in the left fiber endings. Acute pandysautonomia was confirmed after the exclusion of differential diagnoses. Despite the use of intravenous immunoglobulin (2 g/kg), the patient's symptoms did not resolve and he developed chronic dysphagia-related malnutrition. Subsequently, the patient began taking oryzanol, mecobalamin, and lorazepam as a treatment for acute pandysautonomia; this treatment led to slight improvements in symptoms (e.g., vomiting).

**Figure 1:**
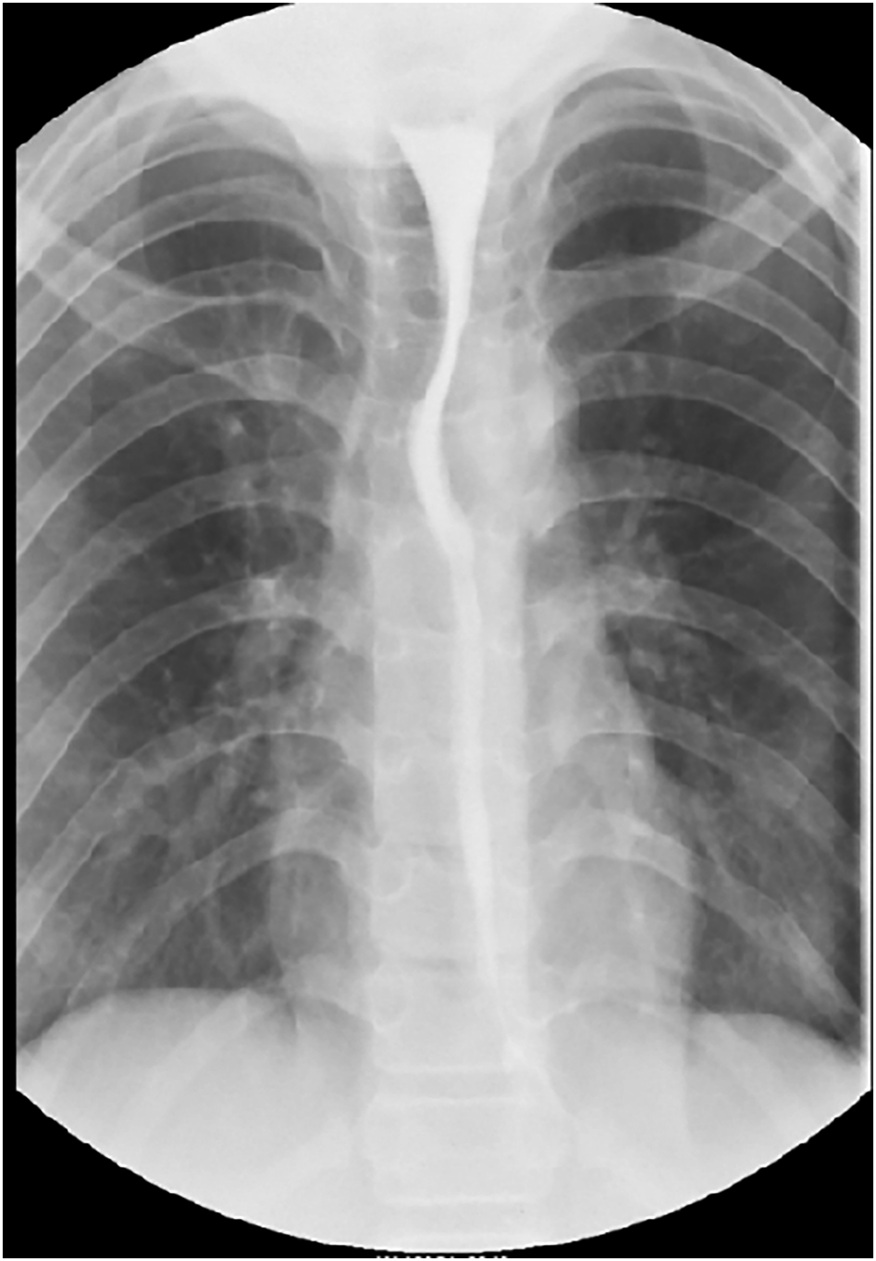
Upper gastrointestinal radiography showed diffusing contraction of the lower esophagus.

**Figure 2:**
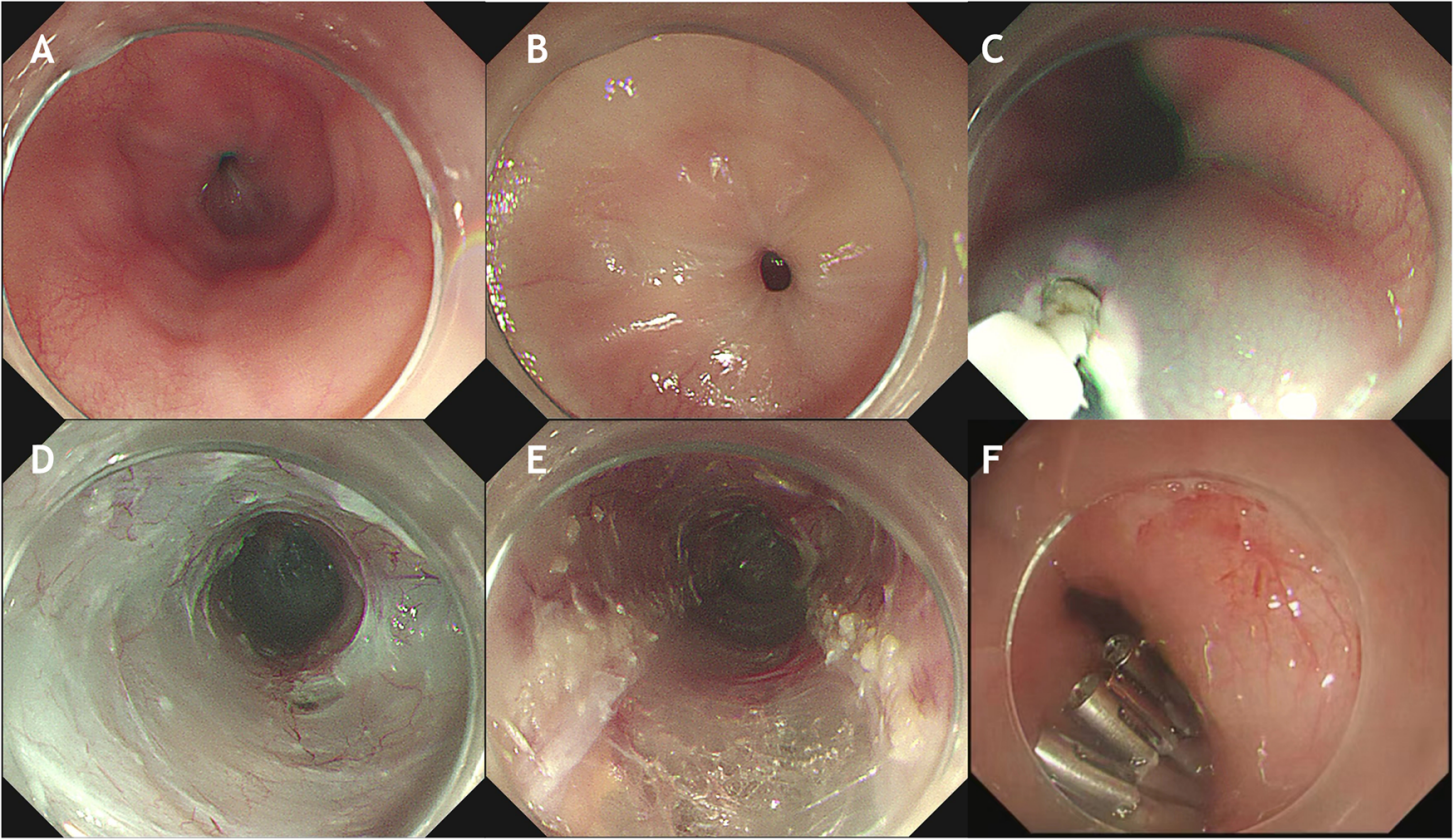
Upper gastrointestinal endoscopy and the technique of POEM in a case with acute pandysautonomia-associated distal esophageal spasm. (**A**) Endoscopic view before POEM revealing contractile ring at the lower esophagus. (**B)** Closed cardiac. (**C**) A longitudinal incision is made into the upper esophagus. (**D**) A submucosal tunnel from the esophagus to the gastric cardia is created. (**E**) Full-thickness myotomy of the lower esophagus. (**F**) Closure of the mucosal incision.

**Figure 3:**
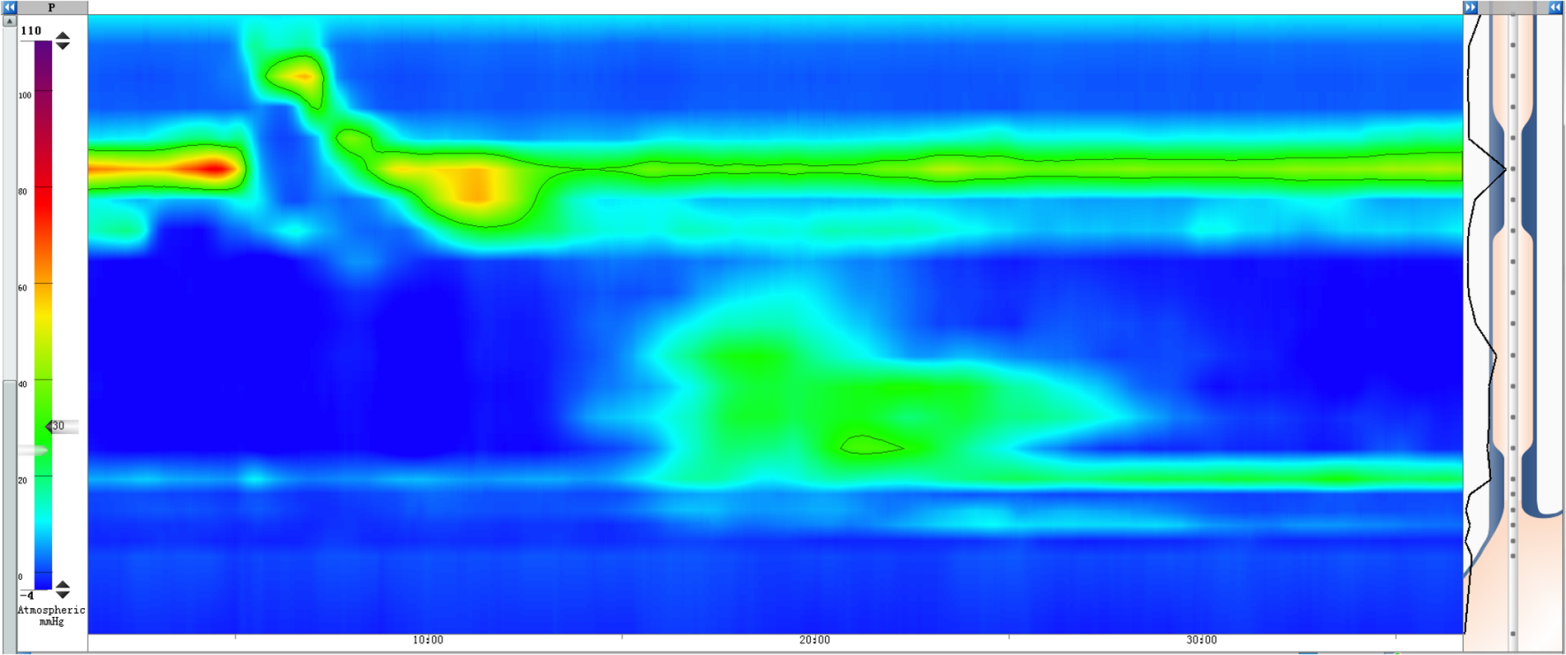
HREM showed a mean IRP of 9.0 mmHg, DCI of 125 mmHg · s · cm, DL of 4.2 s, and a CFV of more than 100 cm/s in test swallows with poor peristalsis in the esophageal body. DCI, distal contractile integral; DL, delayed latency; CFV, contractile frontal velocity.

After admission, the patient continued to exhibit severe dysphagia, postprandial vomiting, and symptoms of autonomic nervous dysfunction, with a baseline Eckardt symptom score of 9 points (dysphagia, 3 points; regurgitation, 3 points; chest pain or discomfort, 2 points; weight loss, 1 point). Abdominal examinations showed normal bowel sounds, no palpable masses, and no signs of organomegaly. Laboratory studies revealed no abnormalities. Upper gastrointestinal radiography demonstrated a diffuse contraction in the lower esophagus (Figure 1). Endoscopic assessment before POEM revealed a contractile ring in the lower esophagus (Figure 2A). Although HREM suggested a mean integrated relaxation pressure of 9.0 mmHg, a distal contractile integral of 125 mmHg·s·cm, delayed latency of 4.2 s, and contractile frontal velocity of >100 cm/s, it did not show evidence of simultaneous contraction (Figure 3).

To relieve the patient's gastrointestinal symptoms since his family refused the treatment of plasmapheresis, POEM was performed under general anesthesia (Figure 2). A tunnel was created using endoscopic submucosal dissection, and proximal-to-distal full-thickness myotomy was performed over a length of 11 cm (25–36 cm from incisors) while preserving the longitudinal muscles. Finally, the mucosal incision was closed using clips. The surgical duration was 40 min. There were no postoperative complications except for subcutaneous emphysema, which was resolved within 2 weeks.

Six months after the procedure, the patient's Eckardt symptom score had decreased to 2 points, he could ingest semi-solid food, and his height and weight increased by 1.5 cm and 2 kg, respectively. Notably, his neurological problems partially persisted.

## Discussion

Acute pandysautonomia is an idiopathic, acute, or subacute type of autonomic neuropathy with unclear etiology and pathogenesis, which has rarely been identified in children (12). There is some evidence that gastrointestinal dysfunction is the most common initial symptom, and symptomatic treatment often produces unsatisfactory curative effects (3). DES is a rare esophageal motility disorder characterized by episodes of dysphagia and/or chest pain (13). Thus far, the relationship between acute pandysautonomia and DES has not been clarified. We have described a 12-year-old boy with acute pandysautonomia-associated DES. Our findings suggest that the digestive symptoms of acute pandysautonomia contributed to the onset of DES in our patient, with progressive symptoms of dysphagia that led to moderate malnutrition.

Acute pandysautonomia is one of the clinical variants of Guillain-Barrè syndrome (GBS) (14). In patients with acute pandysautonomia, early onset and progression of orthostatic hypotension or urogenital dysfunction, urinary incontinence, generalized anhidrosis, and/or laryngeal stridor are highly suggestive of multiple system atrophy (15). In recent years, there have been few reports on the differential diagnosis between autonomic dysfunction and GBS, and the relevant descriptions are not clear. Therefore, more studies are needed to fill in the gaps to guide the diagnosis and treatment of autonomic dysfunction and GBS. In our case, after performing other differential diagnostic tests, we first considered that the patient had acute pandysautonomia.

According to Chicago classification version 4.0, DES is defined as contractions with a distal latency < 4.5 s in the presence of a distal contractile integral of >450 mmHg·s·cm (16). Our patient's HREM findings indicated a distal contractile integral in the range of 100–450 mmHg·s·cm, which did not fully support a diagnosis of DES. However, considering the intermittent nature of the disorder (13), and the finding that HREM did not cause discomfort in the patient, we presumed that the patient was in an intermittent period of DES during the HREM examination, and no data could be collected regarding simultaneous contraction. The findings of ancillary tests (e.g., gastrointestinal angiography and endoscopy) are not specific to DES, but they can provide important evidence to support such a diagnosis (17). Considering the characteristic symptoms and the barium swallow examination findings, we finally diagnosed the patient with DES.

Currently, there are no standard guidelines or consensus recommendations for the treatment of DES. Most medications provide limited symptomatic treatment (18). Recently, POEM has been adopted as an effective treatment for DES because of its excellent short-term clinical outcomes. POEM has also resulted in a significantly higher treatment success rate at 2 years (19–21). Compared with an extended myotomy, POEM is a more targeted procedure, is minimally invasive, and can help to control symptoms in patients with spastic esophageal disorders (22). Multiple studies have demonstrated that POEM is effective (success rate > 80%) and safe for DES, with few significant adverse events (17, 23). According to multiple case reports, POEM results in a satisfactory short-term clinical response in patients with DES (24, 25). Here, we described the successful use of POEM for the treatment of acute pandysautonomia-associated DES in a child. Our findings suggest that POEM is an alternative and promising therapy for acute pandysautonomia-associated DES, but further studies are needed to explore its long-term effects.

## Conclusion

To our knowledge, this is the first report of DES in a child with acute pandysautonomia, and our findings may explain the gastrointestinal symptoms in such patients. POEM is a clinically effective therapeutic method for acute pandysautonomia-associated DES.

## Data Availability

The datasets presented in this study can be found in online repositories. The names of the repository/repositories and accession number(s) can be found in the article/Supplementary Material.
